# Antioxidants in Kidney Diseases: The Impact of Bardoxolone Methyl

**DOI:** 10.1155/2012/321714

**Published:** 2012-06-04

**Authors:** Jorge Rojas-Rivera, Alberto Ortiz, Jesus Egido

**Affiliations:** Laboratory of Renal and Vascular Pathology, Division of Nephrology and Hypertension, IIS Fundación Jiménez Díaz, Autonoma University of Madrid, 28040 Madrid, Spain

## Abstract

Drugs targeting the renin-angiotensin-aldosterone system (RAAS) are the mainstay of therapy to retard the progression of proteinuric chronic kidney disease (CKD) such as diabetic nephropathy. However, diabetic nephropathy is still the first cause of end-stage renal disease. New drugs targeted to the pathogenesis and mechanisms of progression of these diseases beyond RAAS inhibition are needed. There is solid experimental evidence of a key role of oxidative stress and its interrelation with inflammation on renal damage. However, randomized and well-powered trials on these agents in CKD are scarce. We now review the biological bases of oxidative stress and its role in kidney diseases, with focus on diabetic nephropathy, as well as the role of the Keap1-Nrf2 pathway and recent clinical trials targeting this pathway with bardoxolone methyl.

## 1. Background

Chronic kidney disease (CKD) is a serious public health problem, which carries a high morbidity and mortality [1]. CKD is characterized by a progressive loss of renal function, chronic inflammation, oxidative stress, vascular remodeling, and glomerular and tubulointerstitial scarring. CKD treatment still represents a clinical challenge. Diabetic nephropathy (DN) is the leading cause of CKD and end-stage renal disease (ESRD) [2]. The renin-angiotensin-aldosterone system (RAAS) is a major pathway involved in the pathogenesis and progression of DN [3, 4], and RAAS blockade is an effective therapeutic strategy to reduce proteinuria and slow progression of diabetic and nondiabetic CKD. However targeting the system sets off compensatory mechanisms that may increase angiotensin II, aldosterone, or renin, and partial RAAS blockade does not prevent progression in all CKD patients. Angiotensin II (AT II) is the key mediator of the RAAS [5–7]. Animal models of experimental diabetes, clinical trials, and metanalysis have clearly demonstrated the effectiveness of angiotensin-converting enzyme inhibitors (ACEIs) or angiotensin receptor blockers (ARBs) therapy to improve glomerular/tubulointerstitial damage, reduce proteinuria, and decrease CKD progression, independently of blood pressure (BP) control [8–13]. Dual RAAS blockade with ACEI plus ARB inhibits compensatory AT II activity resulting from ACE-independent pathways and limits compensatory AT production induced by AT1 receptor blockade. This combination reduced proteinuria by 25–45% in DN [14–16]. Results are worse for DN with diminished kidney function or nonproteinuric CKD with ischemic renal injury, probably due to advanced structural renal changes [13, 17, 18] and adverse effects; such acute deterioration of renal function or hyperkalemia is more frequent. The aldosterone antagonists spironolactone and eplerenone reduce albuminuria by 30–60% and slow CKD progression in experimental models [19–21] and clinical studies [22–25] in DN. These agents abrogated the “aldosterone breakthrough” phenomenon and its proinflammatory and profibrotic effects. ACEI/ARB therapy increases renin. Aliskiren, a direct renin inhibitor, was beneficial in animal models of diabetic/hypertensive nephropathy [26, 27] and reduced albuminuria in clinical DN [28]. In a multicenter and double-blind, randomized clinical trial in hypertensive type 2 DM patients with nephropathy, aliskiren plus losartan at maximal dose was 20% more effective than losartan/placebo to reduce albuminuria without adverse effects, independent of BP control [29].

A number of other strategies have been tried. Adequate BP and glucose control are part of standard care of DN patients. Intensive glucose control has more impact on GFR if early instituted in patients with type 1 DM but this may not necessarily apply to patients with type 2 DM or with advanced CKD [30]. A trial of the vitamin D activator paricalcitol missed the primary endpoint of albuminuria reduction in DN and caused a transient decrease in eGFR [31]. The nephroprotective effect of statins on CKD found in experimental models has not been conclusively proven in clinical studies [32]. A 1-year dose-ranging study of pirfenidone suggested better preservation of eGFR by pirfenidone in a small number of diabetic nephropathy patients [33]. The selective endothelin antagonist atrasentan reduced albuminuria in a short-term (8 weeks) study in a small number of diabetic patients while receiving RAS inhibitors but did not assess long-term renal function [34]. Heart failure patients or with peripheral edema were excluded.

In spite of all this experimental and clinical evidence, there are 35–40% of patients with DN that progress to advanced renal disease or ESRD. The risk of progression to ESRD is still clinically relevant in other proteinuric nephropathies [35, 36]. Novel therapeutic targets are needed in CKD that are based on a clear understanding of the pathogenesis of CKD progression beyond the RAAS.

## 2. Oxidative Stress and Kidney Disease

 Oxidative stress and inflammation promote kidney and vascular injury [37–42]. Several factors induce ROS in renal cells, such as inflammatory cytokines, Toll-like receptors, Angiotensin II, bradykinin, arachidonic acid, thrombin, growth factors, and mechanical pressure. NADPH oxidases, now renamed Nox enzymes, are key ROS generators in response to these stimuli [43, 44].

 In acute kidney injury (AKI) induced by *ischemic-reperfusion injury*, sepsis or acute rejections ROS contribute of to endothelial and tubular injury [45, 46]. In murine models of AKI, bardoxolone methyl decreased functional and structural renal injury and increased the expression of protective genes (Nrf2, PPAR*γ*, HO-1) on glomerular endothelium, cortical peritubular capillaries, tubules, and interstitial leukocytes [47].

**Figure 1:**
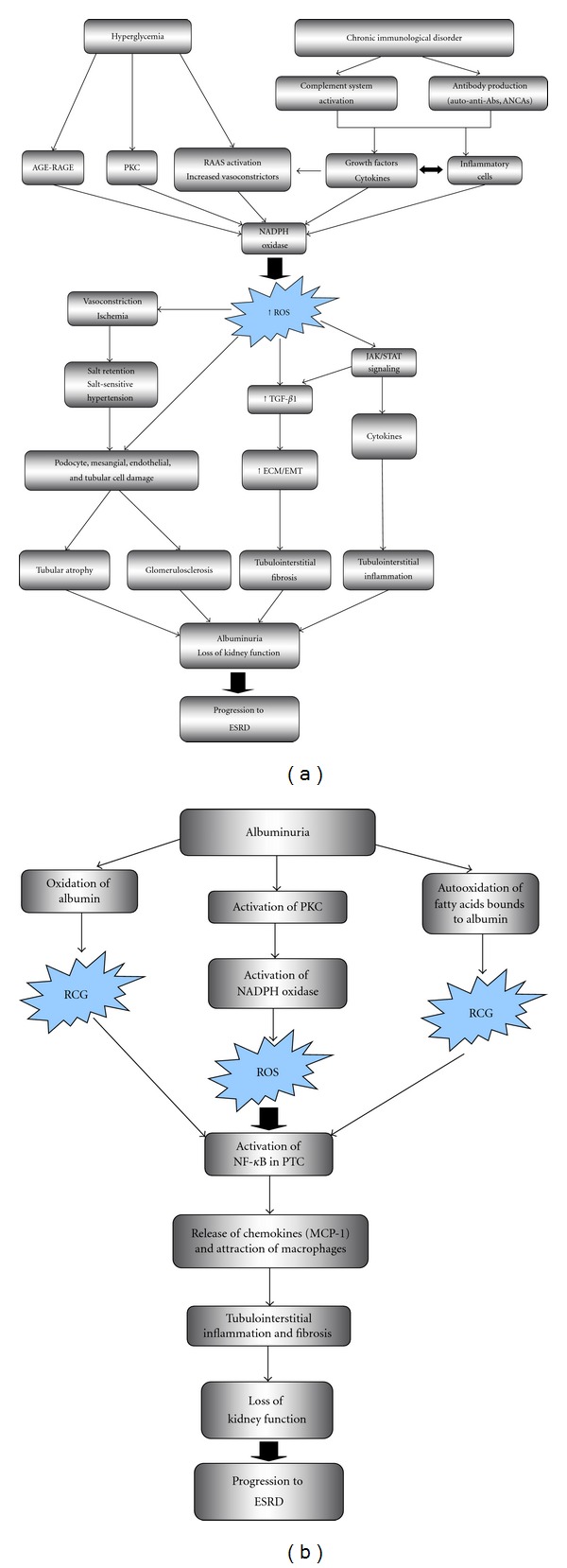
Overview of interrelation of ROS with other key pathogenic factors in kidney disease. (a) Role of ROS in diabetic nephropathy and immune-mediated glomerulonephritis. ROS are induced in renal cells in response to high glucose, AGE, and cytokines. PKC, NADPH oxidase, and mitochondrial metabolism are key to ROS generation. ROS activate signal transduction cascade and transcription factors, leading to upregulation of genes and proteins involved in renal cell injury, glomerular and interstitial extracellular matrix deposition, and recruitment of inflammatory cells, promoting albuminuria and progression of chronic kidney disease. (b) Role of albuminuria and ROS in tubular damage and progression of CKD. Albuminuria injures PTC and activates them to release chemokines that attract macrophages and promote tubulointerstitial fibrosis. Membrane NADPH oxidase is the main source of the ROS. It is possible the generation of other reactive species, as carbonyl groups derived from abnormal oxidation of albumin and fatty acids bound to albumin. Abs: antibodies, AGE: advanced glycation end products, ANCA: antineutrophil cytoplasmic antibodies, ECM: extracellular matrix, EMT: epithelial-mesenchymal transition, ESRD: end-stage renal disease, MCP-1: monocyte chemoattractant protein-1, NADPH: nicotinamide adenine dinucleotide phosphate, NF-kappa B: nuclear factor kappa B, PKC: protein kinase C, PTC: proximal tubular cell, RAAS: renin-angiotensin-aldosterone system, ROS: reactive oxygen species, RCG: reactive carbonyl groups, TGF-*β*1: transforming growth factor beta 1.

 ROS contribute to *hypertension*-induced kidney and vascular injury [41, 43, 48, 49]. The chronic complications of diabetes are characterized by a defect in Nrf2 signaling and its adaptive response to oxidative stress. This is a potential mechanism for cellular stress hypersensitivity and tissue damage [50]. *Diabetic nephropathy* is characterized by initial hyperfiltration, albuminuria and subsequent loss of renal function, thickening of basement membranes, expansion of mesangial matrix and interstitial fibrosis, and podocytes and renal cell damage [51]. ROS production in response to hyperglycemia, protein kinase C (PKC), advanced glycosylation end products (AGEs), free fatty acids, inflammatory cytokines, and TGF-beta1 contributes to these changes [43, 44, 52–54]. These stimuli activate the NADPH/NADPH oxidase system in renal cells. Oxidative stress induced by hyperglycemia or glucose degradation products may cause leukocyte or renal cell apoptosis and release of extracellular matrix [52, 55–59]. PKC activates NF-kappaB, extending the inflammatory response [60]. TGF-beta signaling is key to the excessive matrix formation [61, 62]. The activation of Nrf2 is increased in diabetic nephropathy and can ameliorate mesangial damage via partial inhibition of TGF-beta1 and reduction of extracellular matrix deposition [63]. ACE inhibitors lower TGF-beta in urine from DN patients. In rat DN glomerular HO-1 is increased, evidencing oxidative stress [52, 64]. ROS can activate several transcription factors such as NF-kappaB, AP-1, Sp1, which in turn affect the expression of mediators of inflammation, fibrosis, and cell death [52, 65] (Figure 1(a)).

 ROS also contribute to renal injury in *experimental glomerulonephritis*. In experimental anti-Thy 1 glomerulonephritis, ROS enhance cell proliferation and matrix accumulation and fibrosis and this is improved by the antioxidant alpha-lipoic acid [66, 67]. ROS also regulate the immune response [68]. In nephrotoxic nephritis, neutrophils promote glomerular TNF-alpha expression via H_2_O_2_ production [69]. TNF-alpha is a key mediator of glomerular injury [70]. Interstitial inflammatory leukocytes in proliferative glomerulonephritis locally generate ROS and contribute to sodium retention [71]. Angiotensin II promotes ROS-mediated F-actin cytoskeleton rearrangement, resulting in podocyte injury [72]. In cultured podocytes AT1R signaling activates Rac-1 and NADPH oxidase to produce additional ROS and downregulates the antioxidant protein peroxiredoxin (Prdx2) [73]. In experimental *passive Heymann nephritis*, a model of membranous nephropathy, C5b-9 activation promotes ROS-mediated injury in glomerular cells [74, 75]. In this regard, evidence for oxidative stress, the glomerular neoexpression of aldose reductase (AR) and SOD2, and the appearance of anti-AR and anti-SOD2 autoantibodies was recently reported in human membranous nephropathy suggesting that oxidative stress may generate new autoimmune targets [76].

In *lupus nephritis*, multiple abnormalities in T and B cells lead to autoimmune renal inflammation and ROS production [77]. Nrf2-knockout mice showed impaired antioxidant activity, increased oxidative stress, and a lupus-like autoimmune nephritis with glomerular injury, impaired kidney function, and a shortened lifespan. Thus, Nrf2 deficiency could lead to systemic autoimmune inflammation with enhanced lymphoproliferation [78]. In antineutrophil cytoplasmic antibodies (*ANCAs*-)* associated vasculitis*, ANCA-activated neutrophils and monocytes release MPO and generate ROS, producing endothelial and tissue damage [79, 80]. ANCAs also promote ROS-dependent dysregulation of neutrophil apoptosis [81] (Figure 1(a)).

In *proteinuric nephropathies* and independently of etiology, the presence of albumin in urine activates proximal tubules to release chemokines that promote interstitial inflammation [82]. Albumin uptake by tubular cells activates PKC and ROS generation via NADPH oxidase which activates NF-kappaB and production of inflammatory mediators [83, 84] (Figure 1(b)).

*Accelerated atherogenesis* is common in early and advanced stages of CKD [40, 85]. Oxidative stress contributes to the Immune inflammation-Renal injury-Atherosclerosis complex (IRA paradigm), a condition present in AKI and in CKD, and accelerated atherogenesis [86, 87].

A simplified overview of several components of oxidative stress and its interrelations with other key elements of pathogenesis and progression in kidney disease is shown in Figure 1(a).

## 3. Oxidative Stress and the Keap1-Nrf2 Pathway

 Reactive oxygen species (ROS) include *superoxide anion (SOA), hydrogen peroxide (H_2_O_2_),* and *hydroxyl radical*. ROS are formed continuously as by-product of aerobic metabolism. Sources of ROS include the mitochondrial electron transport chain, metabolism of arachidonate by cyclooxygenases or lipoxygenases, cytochrome P450 enzymes, NADPH oxidases, or nitric oxide synthetases [88]. ROS contribute to killing bacteria, and genetic defects of NADPH oxidase cause chronic granulomatosis [89]. However, ROS may cause chemical damage to DNA, proteins, and unsaturated lipids and lead to cell death. ROS contribute to multiple pathologic processes [48, 90]. In this regard, homeostasis is maintained through a complex set of antioxidants mechanisms that prevent oxidative stress-induced injury. The main mechanisms are enzymes that catalyze antioxidant reactions: *glutathione peroxidase*, *superoxide dismutase*, *catalase*, *Hem-oxygenase (HO-1)*, *NADPH-quinone oxidoreductase* and *glutamate-cysteine ligase*. These enzymes are encoded by stress-response genes or phase 2 genes that contain antioxidant response elements (AREs) in their regulatory regions [88, 91]. Nrf2 is the principal transcription factor that binds to the ARE promoting transcription. Actin-tethered Keap1 is a cytosolic repressor that binds to and retains Nrf2 in the cytoplasm, promoting its proteasomal degradation. Inducers of phase 2 genes modify specific cysteine residues of Keap1 resulting in conformational changes that render Keap1 unable to repress Nrf2. Consequently, Nrf2 activates the transcription of phase 2 genes. Oleanolic acid activates the ARE-Keap1-Nrf2 pathway, resulting in reduced proinflammatory activity of the IKK-beta/NF-kappaB pathway, increases production of antioxidant/reductive molecules, and decreases oxidative stress, thereby restoring redox homeostasis in areas of inflammation. In various cell lines, this results in inhibition of proliferation, promotion of differentiation and apoptosis induction [91–93]. Synthetic analogues of oleanolic acid, named triterpenoids, are potent anti-inflammatory agents that activate the ARE-Keap1-Nrf2 pathway [91]. Bardoxolone methyl, also known as CDDOMe, is a triterpenoid whose nephroprotective action has been recently explored in humans.

## 4. Antioxidant Agents in Kidney Disease

 Epidemiological studies have demonstrated association between inflammatory and oxidative stress markers with cardiovascular and renal outcomes in CKD and ESRD [40, 94–96]. Experimental data in animal models of renal disease suggest beneficial effects of antioxidants agents, but results in human studies are limited and controversial.

 In early experimental diabetes mellitus in hypertensive rats, the administration of tempol, an antioxidant SOD mimetic, corrected the oxidative imbalance and improved oxidative stress-induced renal injury, decreasing albuminuria and fibrosis [97]. Similar protection was afforded by the antioxidants N-acetyl-L-cysteine (thiol) and kallistatin in Dahl salt-sensitive rats [98, 99]. In spontaneously hypertensive rats, a lifelong antioxidant-rich diet diminished the severity of hypertension, improved oxidative stress and ameliorated abnormalities of antioxidant enzyme expressions and activities in contrast to regular diet [100]. In summary, in models of hypertensive rats, synthetic and natural antioxidants induced renal and endothelial protection with reduction of oxidative stress. In a model of ischaemia reperfusion and cyclosporin toxicity after unilateral nephrectomy, the blockage of the mitochondrial enzymes monoamine oxidases with pargyline 28 days following surgery prevented H_2_O_2_ production and improved renal function and renal inflammation (lower IL-1*β* and TNF-*α* gene expression) [101]. Pargyline administrated before ischemia reperfusion significantly reduced apoptosis, necrosis, and fibrosis. This effect was associated to decreased expression of TGF-*β*1, collagen types I, III, and IV and to the normalization of SOD1, catalase, and inflammatory gene expression. In models of renal chronic failure (5/6 nephrectomy rats) [102], AST-120, an oral carbonic adsorbent, improved the oxidative stress in endothelial cells, measured as oxidized/unoxidized albumin ratio. This effect was reached reducing the blood levels of indoxyl sulfate, an uremic toxin that induces ROS. In another model of remnant kidney, the administration of omega-3 fatty acids, an effective compound in mitigating atherosclerosis, significantly lowered several components of oxidative stress and markers of inflammatory and fibrotic response. Furthermore, it attenuated tubulointerstitial fibrosis and inflammation in the remnant kidney [103]. In anti-Thy1 glomerulonephritis, the treatment with parthenolide, an anti-inflammatory agent related to the triterpenoid family, diminished renal inflammation via NF-kappaB inhibition, decreased MCP-1 and iNOS, and improved proteinuria, tubular, and glomerular damage [104]. The beneficial effect of exogenous antioxidants shown in animal models with hypertension or chronic renal failure has not been demonstrated in people with clinical hypertension [96, 105] or CKD.

## 5. Nephroprotection by Bardoxolone Methyl

 Bardoxolone was initially described as an agent that protected cells from radiation-induced damage (radiation mitigator) through Nrf2-dependent and -independent pathways [106]. In humans, its potential antineoplasic activity was evaluated. In phase 1 trials in oncologic patients, bardoxolone unexpectedly improved kidney function, assessed as serum creatinine and creatinine clearance, especially in patients with previous CKD. These findings lead to evaluate potential nephroprotective actions in patients with CKD and type 2 DM, first in an exploratory phase II open-label trial and then in a larger randomized clinical trial. In the first trial [107], 20 patients older than 18 years, with moderate-severe diabetic CKD, were evaluated after 8 weeks of bardoxolone at increasing oral doses of 25 to 75 mg/day. Notably, there was a significant increase in estimated GFR at 4 weeks (+2.8 mL/min/1.73 m^2^) with 25 mg/day and at 8 weeks (+7.2 mL/min/1.73 m^2^) with 75 mg/day. Serum creatinine and BUN decreased and creatinine clearance increased, without changes in total excretion or tubular secretion of creatinine. Unfortunately, GFR was not measured. Blood pressure did not change, and albuminuria had a small albeit not statistically significant increase. Markers of vascular injury and inflammation were improved by treatment with bardoxolone, suggesting a potential beneficial effect on endothelial injury. There were not changes in urine NGAL or NAG adjusted for creatinine concentration, suggesting lack of significant renal toxicity associated to bardoxolone. There were few adverse effects, mainly muscle spasms and a self-limited increase of hepatic enzymes without a true hepatic toxicity. A short followup and an open-label design without control group are major limitations of this study, and they do not allow drawing solid conclusions about the efficacy and long-term safety of this drug on relevant renal outcomes.

 The beneficial effect on eGFR was confirmed in a larger, multicenter, double-blind, randomized trial [108]. This trial randomized 227 patients with moderate-severe CKD and type 2 DM, with stable treatment with ACEI/ARB, to bardoxolone 25, 75 or 150 mg/day or placebo for 52 weeks. Patients were categorized by GFR, urinary albumin-creatinine ratio (UACR), and HbA1c. Patients with hepatic dysfunction or recent cardiovascular events were excluded. A significant improvement in the primary endpoint (change of GFR at 24 weeks) was observed in all bardoxolone groups (+8.2, +11.4 and +10.4 mL/min/1.73 m^2^ resp.) versus 0 mL/min/1.73m^2^ in the placebo group. The secondary endpoint (change of GFR at 52 weeks) also was significantly improved in bardoxolone groups (+5.8, +10.5 and +9.3 mL/min/1.73 m^2^ resp. versus 0). More patients in the placebo group had a GFR decrease ≥25% with respect to baseline value at 24 and 52 weeks. Additionally, serum BUN, phosphorus, and uric acid were significantly lower at 24 and 52 weeks in all bardoxolone groups when compared to placebo.

 Potential unwanted effects included a mild but significant increase of UACR and decreased serum magnesium. UACR increased in patients receiving 75 or 150 mg/day bardoxolone versus placebo. This was observed at 24 and 52 weeks of treatment, but UACR decreased when patients stopped the therapy, suggesting that this effect is reversible. There was an inverse correlation between changes in serum BUN, phosphorus, uric acid, magnesium, and changes in eGFR, as well as a direct correlation between changes in eGFR and changes in UACR, suggesting that changes in eGFR may be the basis for the other observed changes. Interestingly, there was a trend toward higher systolic BP values in the 75 mg bardoxolone group, which was observed despite weight loss and that will merit close attention in further trials. The main adverse effects were muscle spasms (63% of patients in the 75 mg group) and nausea (25%).

 Another significant effect was loss of body weight. This appears to be related to decreased appetite and/or nausea and may be a welcome addition to the therapeutic armamentarium for patients with increased body mass index (BMI). Indeed, weight loss was more evident in patients with higher (>35 kg/m^2^) BMI (mean change −10 kg). However, and perhaps worryingly, it was also observed in patients with normal BMI (−3 kg over 52 weeks).

 The increased eGFR and effects on systolic BP and albuminuria are interesting results on surrogates renal variables which requires more long-term studies. These parameters and, more importantly, cardiac and renal hard end-points (cardiovascular death and progression to ESRD) will be studied at 2 years of followup in an ongoing randomized clinical trial in 1600 patients older than 18 years with advanced CKD (stage 4) and type 2 DM [109]. This study will compare bardoxolone versus placebo in patients receiving standard of care.

## 6. Conclusions and Recommendations

**Figure 2:**
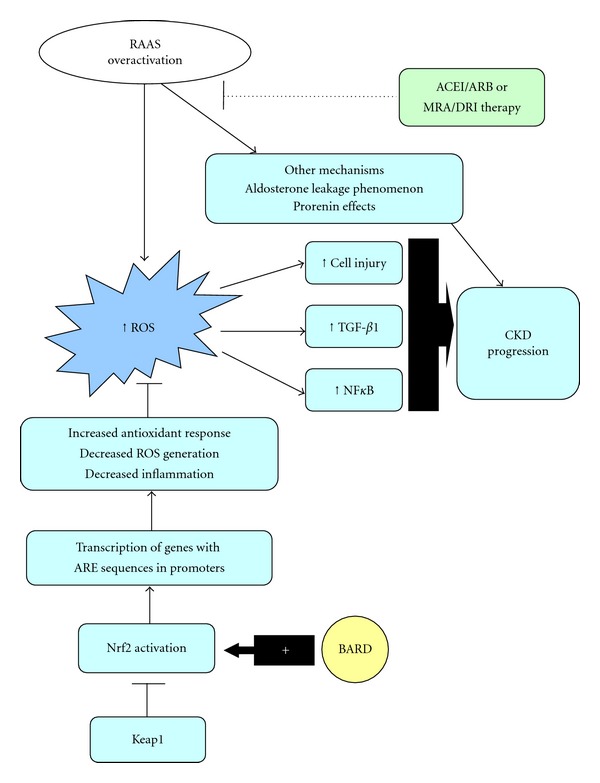
Overview of RAAS blockers and bardoxolone in pathogenic pathways in kidney diseases. RAAS blockers target several pathogenic pathways in kidney injury, including those generating ROS. However, there are several escape mechanisms (aldosterone breakthrough, increased prorenin effects) as well as less sensitive lesions (significant loss of kidney function, ischemic disease, persistent immune activity). BARD promotes activation of the Nrf2 transcription factor, that is released of the inhibitory Keap1 protein and migrates to the nucleus where it regulates transcription of genes containing ARE sequences in their promoters. These phase 2 response genes are collectively involved in the reduction of ROS and inhibition of NF-kappaB. Thus, BARD could promote renal protection through antioxidants and anti-inflammatory effects be promoting the activity of the Nrf2 transcription factor and inhibiting the activity of the NF-kappaB transcription factor. ACE/ACEIs: angiotensin converting enzyme/angiotensin converting enzyme inhibitors, ARBs: angiotensin receptor blockers, AREs: antioxidant response elements, BARD: bardoxolone methyl, CKD: chronic kidney disease, DRI: direct renin inhibitor, mineralocorticoid receptor antagonists, Keap1: Kelch-like ECH-associated protein 1, MRA: mineralocorticoid receptor antagonists, NF-kappaB: nuclear factor kappa B, Nrf2: nuclear factor (erythroid derive 2)-like 2, RAAS: renin-angiotensin-aldosterone system, ROS: reactive oxygen species, TGF*β*-1: transforming growth factor beta 1.

 RAAS blockade is the mainstay of current therapy to slow progression of diabetic and nondiabetic CKD, but this strategy is frequently not enough. Consequently, an important number of patients progress to ESRD. There is solid experimental evidence for a key role of ROS and oxidative stress and their interplay with RAAS and inflammation, in the pathogenesis of CKD. Bardoxolone methyl, a novel synthetic triterpenoid with antioxidant and anti-inflammatory properties, has shown to improve kidney function in patients with advanced DN already receiving RAAS blockers, with few adverse events. This may be a welcomed addition to the therapeutic armamentarium if data are confirmed in larger, longer trials (Figure 2). However, the relative importance and eventual management of the observed influence of bardoxolone on UACR, magnesium, and body weight must be further studied.
